# Platelet distribution width, platelet count, and plateletcrit in diabetic retinopathy: A systematic review and meta-analysis of PRISMA guidelines

**DOI:** 10.1097/MD.0000000000016510

**Published:** 2019-07-19

**Authors:** Shuaifei Ji, Xiaona Ning, Babo Zhang, Heng Shi, Zheng Liu, Jie Zhang

**Affiliations:** a Department of Ophthalmology, Tangdu Hospital; b School of Basic Medicine, The Fourth Military Medical University, Xian, Shaanxi, China.

**Keywords:** diabetic retinopathy, meta-analysis, platelet count, platelet distribution width, plateletcrit

## Abstract

**Background:**

Screening and diagnosis of diabetic retinopathy (DR) mainly depends on fundus examination, which is not an intuitive and simple screening or diagnostic method. Recently, the relationship between platelet parameters and DR has become a hot topic. Whether platelet parameters have clinical value in DR is controversial.

**Methods:**

Literature was retrieved by formal search of electronic databases (PubMed, Embase, Cochrane library, Scopus, and CNKI) and by hand searching of reference lists of related articles from the beginning of building database to December 2017. Review manager 5.3 was utilized to deal with statistical data. This study was registered at International Prospective Register of Systematic Reviews (number: CRD42018093773).

**Results:**

This study included 1720 DR patients, 1477 type 2 diabetic mellitus (T2DM) without DR patients and 1456 health controls in 21 eligible studies. We found there was significant increase of platelet distribution width (PDW) level in the comparison of DR versus Control group (standard mean difference [SMD] [95% confidence interval [CI]] = 1.04 [0.68, 1.40]) and DR versus T2DM without DR group (SMD [95% CI] = 0.68 [0.40, 0.95]). For platelet count (PLT), it showed obvious decrease in the comparison of DR versus T2DM without DR group (SMD [95% CI] = −0.26 [−0.49, −0.03]) and no difference in comparison of DR versus Control (SMD [95% CI] = −0.26 [−0.51, −0.00]). Subgroup analysis showed that significant result of PDW level appeared in China and Turkey in all comparisons, while similar results of PLT only in China. In addition, PDW level was different in various DR-subtypes, obvious high level in proliferation DR.

**Conclusions:**

We concluded that the guiding significance of PDW and PLT in diagnosis and monitor of DR, and especially, application of PDW to PDR management may have potential sense.

## 1 Introduction

Diabetic retinopathy (DR) is one of the most common causes for blindness and vision impairment worldwide, and the proportion of DR-related blindness still is rising. The latest data showed that DR accounted for 3.1% to 4.9% of the blindness burden of Europe (vs 1.1% of world blindness burden).^[1]^ DR is a microvascular complication of diabetic mellitus (DM), a common chronic metabolic disorder, which makes DR more widespread. Nowadays, 3 major treatments of DR are performed, including laser photocoagulation, pharmacotherapy, and vitrectomy, which are effective to reduce vision impairment.^[2]^ However, patients with DR would require long-term treatment and rehabilitation, which lead them suffering from psychological pain and economic burden.

DR is a specific change of ocular fundus abnormalities, characterized by the progressive damage in the retinal microvasculature.^[3]^ It can be classified into proliferation DR (PDR) and nonproliferative DR (NPDR). The stage of PDR is characterized by retinal neovascularization on the optic disc or elsewhere on the retina, which could lead to many complications including retinal detachment, hemorrhage, and glaucoma.^[4]^ The pathophysiology of NPDR is featured with abnormal permeability of retinal capillaries leading to retinal edema, and closure of capillaries leading to retinal nonperfusion and ischemia.^[5]^ Either of them is associated with the microvascular injury and microcirculation disorders of ocular fundus, which indicated that changes of hemorheology may affect the progression of DR.

It is reported that some important cell surface components in the vasculature are altered in a pathological fashion in the hyperglycemic environment during diabetes, which produce the features of progressive DR pathophysiology, including blood-retinal barrier dysfunction, increased expression of inflammatory cell markers, and adhesion of blood leukocytes and platelets.^[6,7]^ Among them, platelet plays an important role in the process of microthrombus formation caused by microcirculation changes, which is a possible pathogenic factor of DR.^[8]^ Platelet distribution width (PDW), platelet count (PLT), and plateletcrit (PCT) are important parameters to reflect the characteristics of platelet. PDW is a marker for measuring the variation of platelet volume and high-level PDW has been reported in diabetic patients recently, especially in these with cardiovascular deceases^[9]^ and microvascular complications in diabetes mellitus.^[10]^ And PLT also was reported to be an independent risk factor for type 2 diabetic mellitus (T2DM) and diabetic nephropathy.^[11]^

At present, the diagnosis of DR still depends on fundus examination, whose applications of early detection and management of DR are relatively limited. Therefore, it is important to look for easier ways to diagnose DR. Although many studies mentioned above have revealed the association of hemostatic and microthrombus abnormalities with T2DM, there were inconsistent conclusions focusing on the relationship between hemostatic parameters and risk of DR. Therefore, the aim of this study was to assess and quantify the differences in PDW, PLT, and PCT comparing subjects with DR, T2DM without DR, and control group, for exploring the clinical prediction of hemostatic parameters for DR.

## 2 Methods

### 2.1 Literature search and identification

Literature was retrieved by formal search of electronic databases (PubMed, Embase, Cochrane library, Scopus, and CNKI) and by hand searching of reference lists of related articles from the beginning of building database to December 2017. The following keywords were used for searching: “diabetic retinopathy,” “platelet distribution width” or “PDW” or “PLT” or “platelet count” or “plateletcrit” or “PCT.” The retrieval strategy of PubMed as follow: (((((platelet distribution width[Title/Abstract] OR PDW[Title/Abstract])) OR (platelet count[Title/Abstract] OR PLT[Title/Abstract] OR (plateletcrit[Title/Abstract] OR PCT[Title/Abstract])) OR platelet[Title/Abstract])) AND (((((Diabetic Retinopathies[Title/Abstract] OR Retinopathies, Diabetic[Title/Abstract] OR Retinopathy, Diabetic[Title/Abstract])) OR Diabetic retinopathy[Title/Abstract])) OR “Diabetic Retinopathy”[Mesh]) Filters: Humans. This systematic review and meta-analysis is reported in accordance with the preferred items for systematic reviews and meta-analysis statement^[12]^ and was registered at the International Prospective Register of Systematic Reviews (number: CRD42018093773). Because this is a secondary study, ethical approval was not necessary.

The following inclusion criteria were adopted for the studies:

1. Published literatures related to the association of PDW or PLT level with DR;
2. Independent case-control studies, cohort study, cross-sectional studies, or randomized controlled trials;
3. The original studies must provide the number of each group and the mean and standard of PDW or PLT;
4. English and Chinese language articles;
5. DR and T2DM patients without other diseases affecting platelet activity, such as cardiovascular disease;
6. DR and T2DM patients did not use anticoagulant or coagulant.

Studies were excluded if:

1. review articles and editorials;
2. case report, animal studies;
3. no-related studies;
4. insufficient data.

### 2.2 Quality assessment and statistical analysis

Study quality was assessed by the Newcastle–Ottawa scale (NOS). Each study was evaluated and scored based on 3 criteria: selection (4 stars), comparability (2 stars), and exposure (3 stars). The NOS point scale ranged from 0 to 9 stars, the researches with NOS ≥7 stars were considered high quality. Two investigators independently assessed the quality of the included studies, and the results were reviewed by a third investigator. Disagreement was resolved by discussion. We utilized Review manager 5.3 to perform the meta-analysis in the present study. Heterogeneity among studies was assessed by *I*^2^ statistic, *P* < .10 and *I*^2^ > 50% indicated evidence of heterogeneity. If heterogeneity existed among the studies, the random effects model was used to estimate the pooled standard mean difference (SMD). Otherwise, the fixed effects model was adopted. The SMD and corresponding 95% confidence interval (CI) was utilized to assess the associations. Subgroup analysis about exploring the relationship between PDW and DR sub-type and the impact of PLT on PDW was performed. Sensitivity analyses by changing effect models were performed to estimate stability of the summary effect. The potential publication bias was investigated using funnel plot.

## 3 Results

### 3.1 Study selection and characteristics

**Figure 1:**
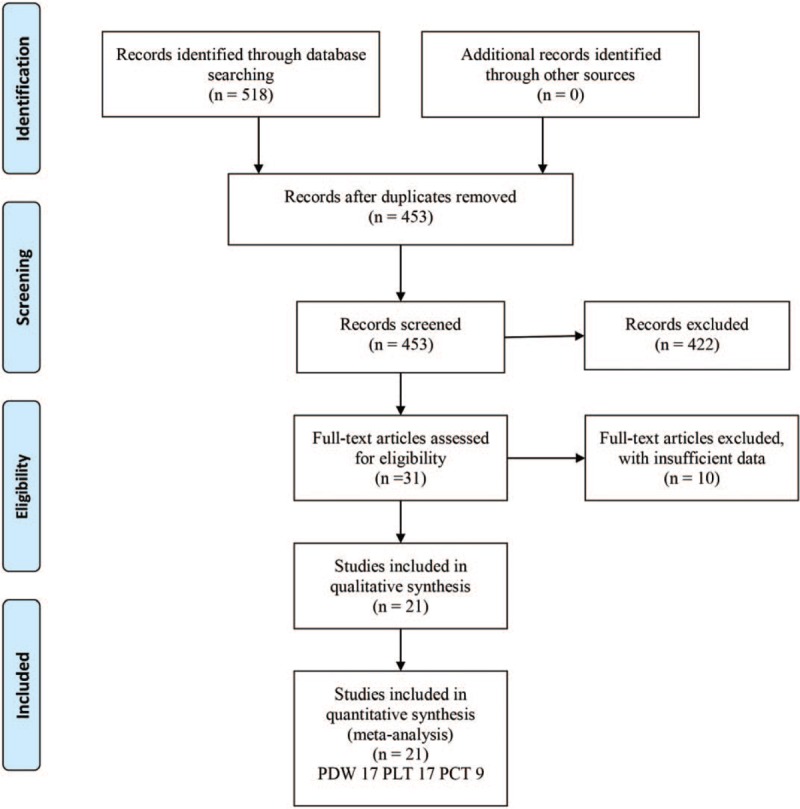
Flow diagram for literature selection.

Based on the search strategy, 21 case-control studies^[13–32]^ from Turkey, China, and India, meet the inclusion criteria and were pooled finally. One study^[32]^ only explore value of platelet parameters in NPDR and PDR, so we include it to perform subgroup analysis about DR sub-type. Quality assessment of all included studies was 6 to 9, and high-quality (≥7) studies accounted for 71.4%. Size of DR patients was from 25 to 174, from 20 to 328 for T2DM without DR and from 20 to 200 for healthy Control. Flow diagram for literature selection was shown in Figure 1, and characteristics of included studies were exhibited in Table 1.

**Table 1:**
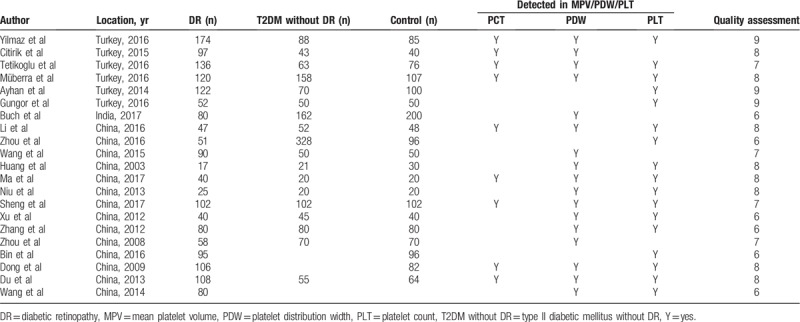
Characteristics of included studies.

## 4 Meta-analysis

### 4.1 Platelet distribution width

**Figure 2:**
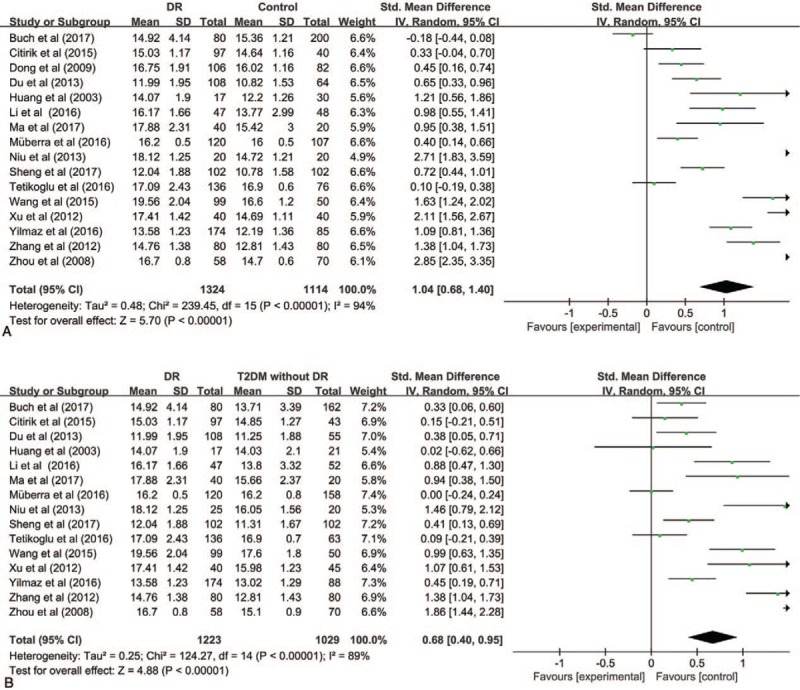
Meta-analysis for platelet distribution width in DR and Control (A) and DR and T2DM without DR (B). DR = diabetic retinopathy, T2DM = type 2 diabetic mellitus.

The results of PDW were summarized in Figure 2. Meta-analysis showed there was significant difference in the Comparison of DR and Control group, the result showed that PDW level in DR group was higher than that in Control group (SMD [95% CI] = 1.04 [0.68, 1.40]) with significant heterogeneity (*P* < .00001, *I*^2^ = 94%). Compared DR with T2DM without DR, the result showed that PDW level in DR group was also higher than that in T2DM without DR group (SMD [95% CI] = 0.68 [0.40, 0.95]) with significant heterogeneity (*P* < .00001, *I*^2^ = 89%). Therefore, the random-effects model was applied to perform meta-analysis.

### 4.2 Platelet count

**Figure 3:**
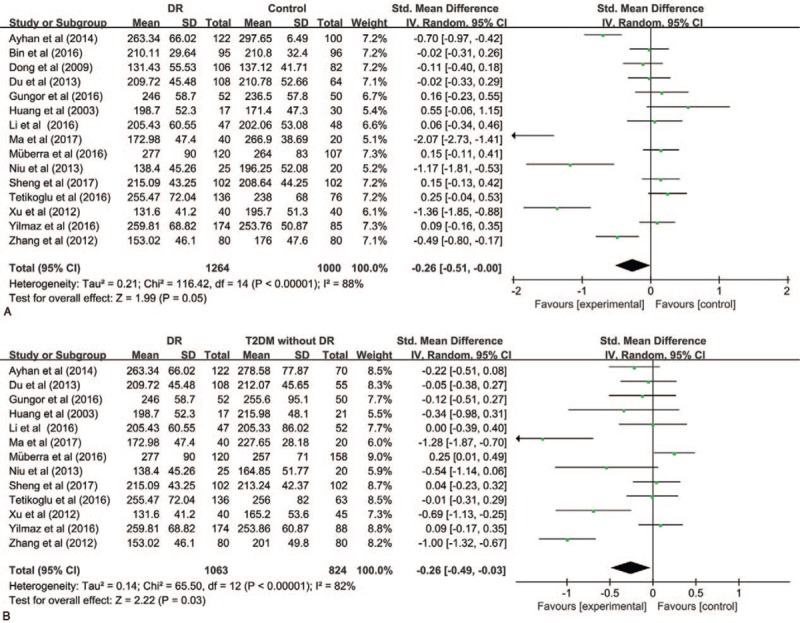
Meta-analysis for platelet count in DR and Control (A) and DR and T2DM without DR (B). DR = diabetic retinopathy, T2DM = type 2 diabetic mellitus.

The results of PLT were summarized in Figure 3. The pooled SMD did not evidence statistically different values of PLT with DR compared to Control group (SMD [95% CI] = −0.26 [−0.51, −0.00]), while, compared to T2DM without DR, PLT level decreased in DR (SMD [95% CI] = −0.26 [−0.49, −0.03]). *I*^2^ test indicated that the heterogeneity was significant (*P* < .00001, *I*^2^ = 88%, *I*^2^ = 21%). Therefore, the random-effects were applied to perform meta-analysis.

### 4.3 Plateletcrit

**Figure 4:**
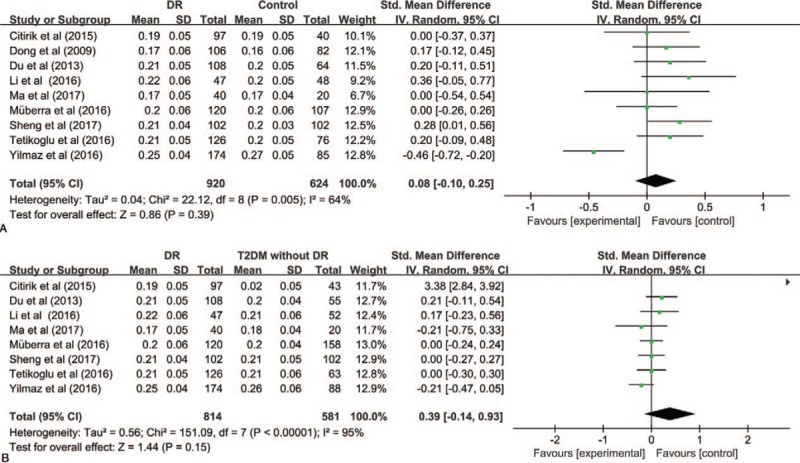
Meta-analysis for platelet count in DR and Control (A) and DR and T2DM without DR (B). DR = diabetic retinopathy, T2DM = type 2 diabetic mellitus.

As shown in Figure 4, there was no significant difference of PCT with DR compared to Control group (SMD [95% CI] = 0.08 [−0.10, 0.25]) and T2DM without DR (SMD [95% CI] = 0.39 [−0.14, 0.93]). Given obvious heterogeneity, likewise, random-effects were performed.

### 4.4 Subgroup analysis

Subgroup analysis was performed to explore the differences of PDW and PLT in country and subtype (Table 2). We discovered that, no matter of DR versus Control and DR versus T2DM without DR, PDW exhibited significant differences in both China and Turkey, while similar results of PLT only in China. In DR subtype, evidences of PDW also in PDR versus Control (SMD [95% CI] = 0.73 [0.24, 1.22]), PDR versus T2DM without DR (SMD [95% CI] = 0.49 [0.11, 0.88]) and PDR versus NPDR (SMD [95% CI] = 0.28 [0.02, 0.54]), but PLT did not showed any difference in subtype.

**Table 2:**
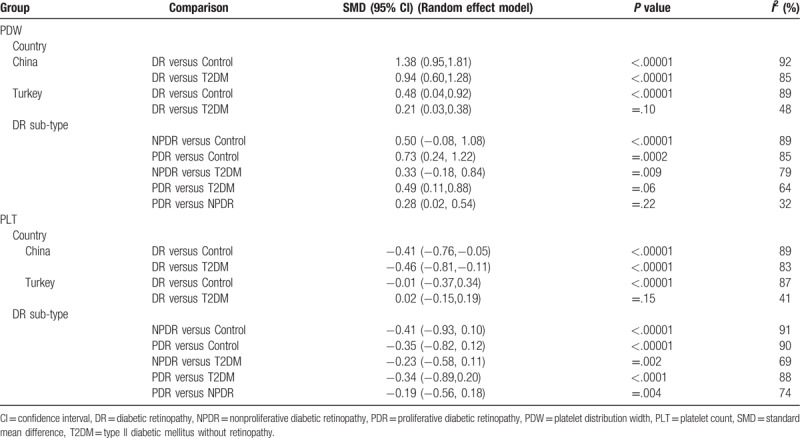
Summary of standardized mean differences among subgroups.

### 4.5 Sensitive analysis and publication bias

**Figure 5:**
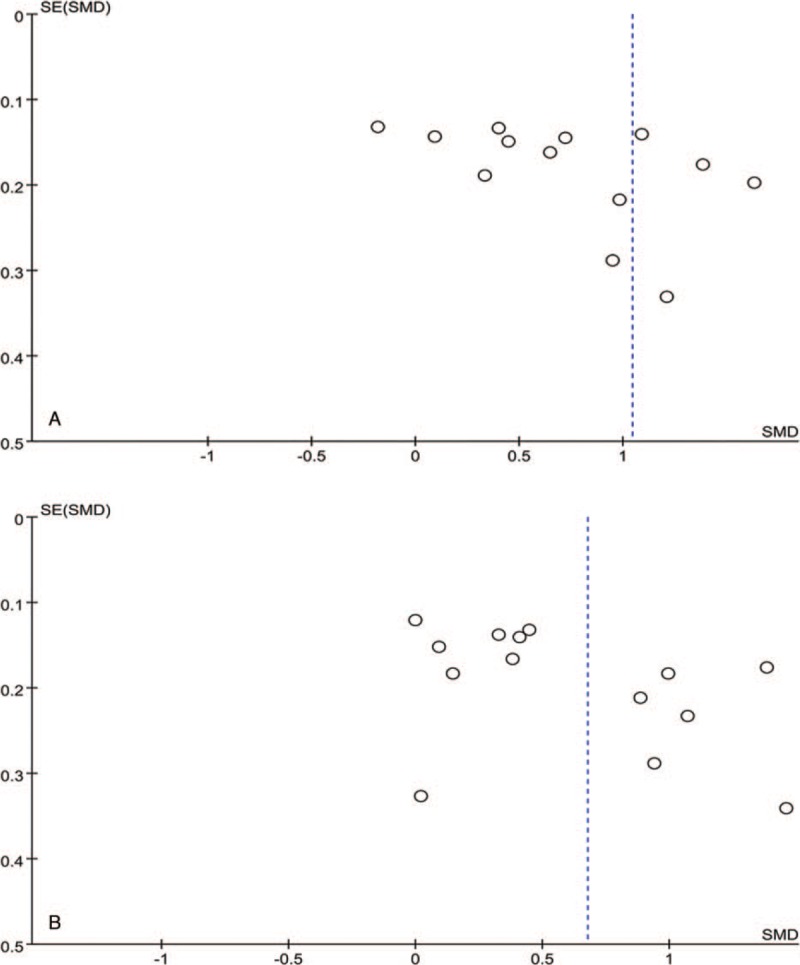
Funnel plots for the difference of PDW in DR versus Control (A) and DR versus T2DM without DR (B). DR = diabetic retinopathy, PDW = platelet distribution width, T2DM = type 2 diabetic mellitus.

Sensitive analysis was analyzed by changing effect model, and results suggested all of the summary effect didn’t change significantly, except for PLT in DR vs Control [SMD(95%CI) = −0.31 (−0.21,−0.04)] (Table 3). Results of publication bias were shown in Figure 4, and funnel plots exhibited good symmetry (Figure 5). Given small-sized studies included, we didn’t conduct publication bias about PLT and PCT.

**Table 3:**
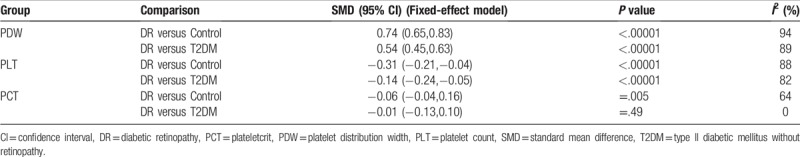
Sensitive analysis about the contribution of single study on the summary results.

## 5 Discussion

DM is a growing health problem and accompanied by a high risk of vascular complications, of which DR is the leading cause of blindness in the working-aged population in the United States.^[33]^ We keep hope to prevent blindness due to DR via adequate screening with technology. Studies have shown that some changes of hemorheology would occurred in DM, especially PLT parameters, which leads to pathological changes of blood vessels.^[10,11,34,35]^ As 2 important parameters of platelet, PDW and PLT attracted researchers’ attention and different opinions about whether they differ between patients and healthy controls were proposed. Thus, we systematically reviewed and summarized through a meta-analysis to explore the relationship between platelet parameters and DR, for identifying biomarkers for early detection of diabetic complications.

Studies showed that increased procoagulant factors and tissue factor associated with impaired fibrinolysis, platelet hyperreactivity, endothelial dysfunction, leukocyte activation, low-grade inflammation, and microparticle involvement, they all play a role in the establishment of this prothrombotic condition.^[7]^ Changes in hemorheology derived from these factors lead to the occurrence of diabetic complications. For DR, the risk factors can be inducted to hyperglycemia, hypertension, dyslipidemia, and diabetes duration.^[36]^ Studies considered that the most notable reactome pathway of DR was “platelet degranulation.”^[37]^ In addition, procoagulant activity in DR patients may be partly ascribed to phosphatidylserine exposure and microparticles release from blood and endothelial cells.^[38]^ Thus, platelets contribute cooperatively to the hypercoagulable state of DR patients and play an important role in formation of DR.

Platelets are the smallest cells in the blood and hold the physiological characteristics such as adhesion, aggregation, and release. They participate in the hemostasis and coagulation process of the human body and maintain the integrity of the blood vessel wall. Increased activation and aggregation of platelets are important causes of vascular complications in diabetes.^[8]^ In our study, there were no differences between DR and control group in PLT level, which was consistent with other studies,^[39,40]^ while compared with T2DM without DR, PLT level decreased significantly. We conjectured that consumption during coagulation mainly attributes to decreased PLT in DR patients. In addition, PCT, the percentage of platelets in blood per unit volume, exhibited no evidence in the comparisons, no matter of DR versus Control and DR versus T2DM without DR.

PDW can directly measure the variability in platelet size, and its high values suggest increased production of larger reticulated platelets,^[41]^ which is associated with thrombotic formation. In the pooled analysis of PDW, we reached the conclusion that high values appeared in DR compared to either T2DM without DR group or healthy control group, and it not only indicated the clinical value of platelet in the direct screening of DR in type II diabetic patients and healthy people, but also that PDW may be associated with the risk of retinopathy of T2DM. In addition, sensitive analysis and publication bias exhibited our results were reliable.

As for subgroup analysis, we found that significant difference of PDW level in the comparisons, which included China and Turkey, but similar results of PLT only appeared in China. Therefore, geographical area may be a potential factor. For DR-type and stage, there was significant difference in the comparisons of PDR versus Control group, versus T2DM without DR group and versus NPDR group. Thus, PDW has the potential to be applied for PDR management. While PLT did not exhibit such significance.

Of course, we need point out some limits in our article. First, the heterogeneity could not be explained completely, even if subgroup analysis was conducted. Second, we could not ensure all of factors match due to case-control studies included. Third, difference caused by geographical area was not clear. Finally, we failed to reveal the reason of results of PCT. Taking account of the limits in this study, more rigorous and high-quality researches need to be implemented to further confirm our conclusions.

In conclusion, our studies indicated that the guiding significance of PDW in diagnosis and monitor of DR, especially PDR, and application of PLT for Chinese DR patients is worthy to be explored. PDW and PLT are easily accessible platelet parameters, so they may be of great significance for monitoring the development and progression of DR.

## Author contributions

**Conceptualization:** Xiaona Ning, Zheng Liu.

**Data curation:** Xiaona Ning.

**Formal analysis:** Zheng Liu.

**Funding acquisition:** Zheng Liu.

**Investigation:** Babo Zhang.

**Methodology:** Babo Zhang.

**Project administration:** Babo Zhang.

**Software:** Babo Zhang.

**Validation:** Heng Shi.

**Visualization:** Heng Shi.

**Writing – original draft:** Shuaifei Ji.

**Writing – review and editing:** Shuaifei Ji, Jie Zhang.
